# Assessment of cognitive dysfunction in traumatic brain injury patients: a review

**DOI:** 10.1080/20961790.2017.1390836

**Published:** 2017-11-14

**Authors:** Huiyan Sun, Chengliang Luo, Xiping Chen, Luyang Tao

**Affiliations:** a School of Biology & Basic Medical Science, Soochow University, Suzhou, China; b Affiliated Hospital, Chifeng University, Inner Mongolia, China

**Keywords:** Forensic science, traumatic brain injury, event-related potential, P300, cognitive impairment

## Abstract

Traumatic brain injury (TBI) is one of the major causes of human mortality and morbidity in the world. Brain injury could affect the core of a person's being – their thinking, memory, personality and behaviour. Electrophysiological markers from the human electroencephalogram and brain imaging provide a rich source of data which helps to elucidate specific processing impairments in TBI patients. To assess the cognitive and social function in traumatic brain injury patients, this review will focus on some of methods for assessing the disabling cognitive and social function deficits induced by TBI. There are many new technologies available to address TBI and recognition related questions. Integration of the various techniques will help to facilitate our comprehending of TBI, cognitive function and social function, and improve treatment and rehabilitation.

## Introduction

### Traumatic brain injury (TBI)

TBI is one of the major causes of human mortality and morbidity in the world, with at least 10 million serious traumatic brain injuries occurring annually [1,2]. TBI is a complex, heterogeneous disorder with many factors, contributing to a spectrum of severity from mild TBI to severe brain injury. From 2000 to 2015, 82% of military members in U.S. sustained a TBI is classified as mild TBI (mTBI) [3]. TBI is not a disease, but it is an event. More precisely, TBI is an event or a sequence of events that can, in some instances, lead to a diagnosable neurological or psychiatric disorder [4].

### Cognitive function and social function

Cognitive function is “the mental action or process of acquiring knowledge and understanding through thought, experience, and senses” [5]. It encompasses processes, including knowledge, attention, memory and working memory, judgment and evaluation, reasoning and “computation”, problem solving and decision-making, comprehension and production of language. Cognitive processes use existing knowledge and generate new knowledge. Cognitive dysfunction is a prominent symptom aftr TBI, including planning, problem solving, temporal organization, attention, cognitive-behavioural and psychobehavioural disorders. Persistent memory impairment and executive function impaired after TBI is also common [6]. Retrograde amnesia persists in patients who survive TBI, similar to cognitive deficits often associated with TBI [7]. Survivors of severe TBI often have cognitive control functions impairments. The TBI patients showed a specific performance deficit suggestive of a failure to implement cognitive control in the service of processing conflict information and detecting response conflict and signalling for recruitment of cognitive resources to properly adjust performance [8,9]. The mTBI is associated with intact conflict monitoring, and also alteres conflict adaptation and adjustment processes [10]. Yu et al. [11] suggest that even very mild mechanical events may lead to a quantifiable neuronal network dysfunction, and mild pediatric TBI could result in functional deficits that are more serious than appearance at present [12].

The injury of the brain can alter the core of a person's being – their thinking, personality and behaviour, especially in the social behaviour [13]. Previous research suggests that such deficits may result from impaired perception of basic social cues [14]. Green et al. [15] reported that teens (15–18 years old) sustained TBI between birth and five years old childhood were more likely to experience poor quality-of-life compared to health controls. The severe TBI patients produce tangential language and have difficulties identifying communication breakdown, asking questions and engaging in conversational joking in casual conversations with friends [16]. Lachapelle et al. [17] evaluate low-level to complex information processing by using visual electrophysiology and examine the prognostic value with regard to vocational outcome in persons who have sustained a mTBI. The findings suggest that individuals with symptomatic mTBI can present selective deficits in intricate visual information processing that could interfere with vocational outcome. There are also impairments in certain aspects of emotion perception: affective state, but not appraisal and regulation, contribute to social behaviour difficulties in severe TBI patients. And it has important implications for rehabilitation [18]. Accident phobia can impede safe return to driving or motor vehicle travel, inhibiting return to daily functioning in mTBI. In addition, pain complaints have been found to correlate positively with postinjury anxiety disorders [19].

It is evaluated that around 0.85 million civilians require long-term rehabilitation and care for the residual of their lives because of a TBI each year in the United States [13]. So there is a growing public and scientific interest in the causes, sequelae and treatment of TBI. Brain injury could affect the core of a person's being – their thinking, memory, personality and behaviour. There are many new technologies available to address TBI and recognition-related questions. Integration of the various techniques will help to facilitate our comprehending of TBI, cognitive function and social function, and improve treatment and rehabilitation. So we select this topic to focus on some of methods for assessing the central and disabling cognitive and social function deficits induced by TBI.

## Assessment methods

There are substantive reviews and empirical papers proposing important questions on reflecting the spectrum of injury severity of survivors of TBI. The approaches include somatic, autonomic and central nervous system psychophysiological ways, and brain imaging (e.g. structural and functional magnetic resonance imaging, fMRI). A literature integrates information across these domains [20]. The articles in this issue thus reflect the multiplicity of contemporary cognitive and social function assessment methods in the patients after TBI.

### Neuropsychological assessment

Neuropsychological assessment was traditionally carried out to assess the extent of impairment to a particular skill and to attempt to determine the area of the brain which may have been damaged following brain injury. It focuses on the assessment of cognition and behaviour, including examining the effects of any brain injury or neuropathological process that a person may have experienced. Neuropsychological testing is more than the administration and scoring of tests and screening tools. It is essential that neuropsychological assessment also include an evaluation of the person's mental status.

### Electrophysiological markers

In neuroscience, electrophysiology includes measurements of the electrical activity of neurons, and in particular, action potential activity. The electrophysiological research comprises electroencephalogram (EEG), sensory evoked potentials (EPs), and cognitive event-related potentials (ERPs). They are useful for electrodiagnosis and monitoring.

EEG is an electrophysiological monitoring method of recording electrical activity of the brain. It is typically noninvasive, with the electrodes placed along the scalp, although invasive electrodes are sometimes used in specific applications. EEG measures voltage fluctuations resulting from ionic current within the neurons of the brain. Because EPs and ERPs are generated by neuronal activity, they are valuable for assessing the integrity of neural processing capabilities in patients sustained TBI [27]. Derivatives of the EEG technique include EPs, which involves averaging the EEG activity time-locked to the presentation of a stimulus of some sort (visual, somatosensory, or auditory). ERPs and oscillatory activity Alpha from the human EEG provides a rich source of data that helps clarify specific processing impairments in TBI patients. Some of the disabling cognitive deficits in TBI and how broadband ERP markers and the spectral content of the EEG contribute to explain abnormalities in brain function that impact upon processing speed, sustained attention, performance monitoring, inhibitory control and cognitive flexibility [21–24]. They refer to averaged EEG responses that are time-locked to more complex processing of stimuli; this technique is used in cognitive science, cognitive psychology and psychophysiological research.

EPs are the most informative neurophysiological tests. Both have the major prognostic value in TBI [25,26]. In TBI, these EPs patterns have high early prognostic value for any outcome [27]. Continuous EEG monitoring is used for diagnosis and treatment of non convulsive seizures and status epilepticus, whereas EPs are more able to indicate the emergence of neurological deterioration. The EEG seems to have the same prognostic value in pediatric as in adult [28]. Recent reviews also supported the use of EPs in the integrated process of outcome prediction after acute brain injury in children [29]. Amantini et al. [30] evaluate the prognostic utility of EPs in severe TBI considering both “awakening” and disability. EPs were able to predict the correct prognosis in more than 80% of severe TBI. They confirm the high predictive utility of EPs in TBI, which is greater than Glasgow coma scale (GCS) and EEG reactivity [30].

ERPs were powerful tools for prognosticating the trajectory of recovery and ultimate outcome from the TBI. Short- and middle-latency EPs can now effectively predict coma outcomes in acute TBI patients. Long-latency ERP components hold promise in predicting recovery of higher order cognitive function [14]. The error-negativity/error-related negativity (Ne/ERN) and post-error positivity (Pe) can be measured. The Ne/ERN was a potential electrophysiological marker of evaluative control/performance monitoring impairment following TBI [31,32]. ERPs were used to elucidate the nature of cognitive complaints of TBI survivors in military populations, emphasizing defect in attention, information processing and cognitive control. The application of ERPs to predict emergence from coma and eventual outcome is also highlighted [33]. Larson et al. [32] exploited the ERN and Pe components of the ERP to test the hypothesis that negative affect disproportionately impairs performance-monitoring following severe TBI. It supports a “double jeopardy” hypothesis of disproportionate impairments in performance monitoring when negative affect is overlaid. Other paper also report in the mTBI. The neural bases of performance monitoring can be detected using the ERN and Pe components of the scalp-recorded ERP in mTBI survivors. Main effects or interactions of group for behavioural and ERP measures were not significant. Also, there were no significant subgroup and correlational analyses with post-concussive symptoms and indices of injury severity [12]. For ERPs, the conflict slow potential, controls showed significant conflict adaptation, whereas individuals with mild TBI did not [8]. An electrophysiological marker of impaired function rewards context sensitivity following severe TBI. The “feedback-related negativity” (FRN) – an ERP component evoked following performance or response feedback with a larger FRN following unfavourable than favourable outcomes [31].

Colours are thought to affect human cognition and emotion. P300 amplitude and latency are valuable indexes for the evaluation of TBI patients, and that colour environments of red, green or darkness affect cognitive function [20,34]. Lew et al. [12] measured the P300 ERP, which has been shown to be a sensitive index of cognitive efficiency. TBI patients showed remarkable impaired electrophysiological and behavioural responses while attempting to detect affective facial cues [16]. Using auditory and visual stimuli (including facial affective stimuli), Doi et al. [35] analysed the P300 components of ERPs in patients after TBI, to assess their cognitive characteristics. Lew and his colleagues compared the effectiveness of P300 ERPs in discriminating patients with TBI from healthy control subjects. There is a remarkably high correlation between duration of posttraumatic amnesia and P300 amplitude [36]. The low P300 amplitude may be used for emotion detection [37]. The latency of N2 and P3 in passive task of ERP can also be used as an indication for evaluating cognitive function in patients with diffuse brain injury [38].

Sarno et al. [39] aimed to investigate attentional resources distribution in TBI patients. They recorded Auditory ERPs and found evidence of deficits in the early stages of information processing and allocation of attentional resources. Analysing the response to standards was crucial for detecting new aspects of attentional impairments [39]. The mismatch negativity (MMN) is a component of the long-latency auditory EPs, and it could check the functionality of automatic attentional processes of attentive information processing. MMN is a useful aid to differentiate vegetative state from minimal conscientious state during the subacute phase of severe TBI [40]. It is arguably one of the most valid predictors in predicting outcome from coma [41].

### Brain imaging methods

Sozda et al. [20] highlighted advances in psychophysical and imaging research in TBI, which could increase our comprehending of the neural mechanisms of cognitive, physical and affective sequelae of TBI.

By using brain imaging methods (e.g. CT, MRI), major data is obtained, which plays an important role in identifying brain injury-related trauma, particularly for moderate-to-severe TBI. The current developments in brain imaging methodologies, for both clinical and research applications, offer considerable promise in improving diagnosis, understanding cognitive impairments and informing treatment and rehabilitation efforts [42]. McAllister and colleagues used fMRI in a unique study to detect the effects of an alpha-2 adrenergic agonist (guanfacine) on working memory and brain activity in mild TBI, and pointed to potential pharmacological interventions to improve cognitive impairment in TBI [43].

FitzGerald and Crosson [44] provided a review of diffusion-tensor imaging (DTI) methods and their application to the study of TBI. DTI can measure injury-related microstructural changes in the brain, and also has been effectively employed to assess relationships between TBI-related symptoms, cognitive performance and changes in brain microstructure and white-matter connectivity. They conclude that DTI has considerable promise as a “biomarker” of mild TBI. It has superior predictive value for neurocognitive out-come relative to conventional neuroimaging [45].

### Genetic polymorphism

It is becoming increasingly clear that genetic factors play a role in the person's cognitive and social function after TBI. Weaver et al. [46] consider the potential for polymorphisms to influence six specific cognitive and social functions after TBI: working memory, executive function, decision-making, inhibition and impulsivity, aggression, social and emotional function. Brain-derived neurotrophic factor (BDNF), a member of the neurotrophin family, is a strong predictor of everyday decision-making, occupational attainment, social mobility and job performance. Two single-nucleotide polymorphisms, rs7124442 and rs1519480, were significantly associated with 30–35 years post-injury recovery of general cognitive intelligence with the most pronounced effect at the time of 10–15 years post-injury, indicating lesion-induced plasticity [52]. The effect of BDNF provided insight into a significant aspect of post-traumatic cognitive recovery and associated with executive functions in TBI subjects [47]. Individuals with the serotonin-transporter-linked polymorphic region (5-HTTLPR) variant short/short genotype have increased sensitivity to both positive and negative perceptions of perceived social support. Veterans with TBI appeared to increase sensitivity to social stress, and the Veterans who were L' allele after TBI fared the worst, with lower resilience and more perceived limitations for community participation contrast to L' carrier Veterans without a TBI or Veterans with the S'S' genotype regardless of TBI status [48]. Response time and accuracy differences were found deficit in persons with TBI, indicating that persons with TBI may have difficulties in processing single words, especially under conditions of increased executive demand [49]. Kurowski et al. [50] study the association of a functional catechol-*O*-methyltransferase genotype (rs4680) with recovery of executive functions after TBI. Stimulation of bradykinin release by activated factor XII probably takes effect on expanding secondary brain damage by promoting brain edema formation and inflammation. The TBI-associated pathologic processes will be alleviated by blocking of activated factor XII [51]. The altered microRNA expression levels in cerebrospinal fluid after TBI together with single nucleotide polymorphisms identified within the microRNA gene promoter area provide a new perspective on the mechanism of impaired consciousness after TBI [52].

## Conclusion

Progress has been made in recent years in processes of cognitive deficits after traumatic brain injury, such as electrophysiological markers and brain imaging methods. There are many new technologies available to address TBI and recognition-related questions. Integration of the various techniques will facilitate our comprehending of TBI, cognitive function and social function, and improve treatment and rehabilitation efforts. Electrophysiological markers with the addition of sensitive laboratory paradigms (especially ERPs) is a reliable method to predict cognitive disturbances after brain injury.
